# A Importância dos Estudos de Evolução Temporal Usando Modelos Experimentais de Doenças Cardíacas

**DOI:** 10.36660/abc.20210997

**Published:** 2022-02-14

**Authors:** Diego Santos Souza, Danilo Roman-Campos

**Affiliations:** 1 Departamento de Biofísica Escola Paulista de Medicina Universidade Federal de São Paulo São Paulo SP Brasil Laboratório de CardioBiologia, Departamento de Biofísica, Escola Paulista de Medicina, Universidade Federal de São Paulo, São Paulo, SP – Brasil

**Keywords:** Doenças Cardiovasculares, Insuficiência Cardíaca, Mortalidade, Custos de Cuidados Médicos, Remodelação Ventricular, Miócitos Cardíacos, Ratos

De acordo com o *American College of Cardiology* , a Síndrome de Insuficiência Cardíaca (IC) é definida como uma síndrome clínica complexa que resulta de qualquer comprometimento estrutural ou funcional do enchimento ventricular ou da ejeção de sangue.^1^ O agente etiológico da síndrome clínica da IC pode ter diferentes origens, as quais impactam no prognóstico e impõem a necessidade de estratégias de tratamento específicas. Deve-se ressaltar a relevância de dados específicos para enfatizar essas diferenças na IC, incluindo sua evolução temporal.^2^ Esta visão é similar à atual diretriz brasileira para IC.^3^ Apesar da melhora no tratamento e gestão da IC, com uma redução na taxa de mortalidade geral ao longo do tempo,^4^ o número de mortes e os custos econômicos ainda são altos. Em 2014, uma revisão profunda, incluindo 197 países, mostrou que o custo econômico global da IC em 2012 foi estimado em US $ 108 bilhões por ano.^5^ É importante ressaltar que os gastos da IC foram muito diferentes em países de alta, média e baixa renda.^5^ Por exemplo, entre 2014-2020, os custos médicos totais medianos anuais para atendimento à insuficiência cardíaca foram estimados em US $ 24.383 por paciente nos Estados Unidos da América,^6^ enquanto no Brasil em 2015, o custo médio por paciente com IC foi de 1569 US $, considerando a taxa de câmbio de 2015.^7^ O alto custo por paciente provavelmente está relacionado à falta de terapias eficazes para melhorar a saúde humana durante a IC. Então, desvendar as bases moleculares da remodelação cardíaca encontrada na doença é um dos principais desafios da medicina cardiovascular.

Nos últimos anos, avanços significativos na compreensão dos mecanismos moleculares da hipertrofia compensada e descompensada e da insuficiência cardíaca em resposta aos sinais de estresse mostraram que muitos fatores extracelulares e vias de sinalização estão envolvidos na remodelação dos cardiomiócitos, as células contráteis funcionais encontradas no tecido cardíaco. Como o curso da IC é etiológico-dependente, é fundamental entender como a síndrome de IC se desenvolve em diferentes modelos. Por exemplo, a dinâmica intracelular do Ca^2^ é modificada nas diferentes etiologias de IC com fração de ejeção reduzida (HFrEF) ou preservada (HFpEF). Em modelos animais de HFpEF e HFrEF diabéticos ou hipertensos, os estudos mostraram que a magnitude e a cinética da liberação de Ca^2^permanecem inalteradas em ambos os modelos de HFpEF, mas prejudicadas em HFrEF. Embora a densidade da proteína Sarco/retículo endoplasmático Ca^2^ adenosina trifosfatase-2a (SERCA) tenha sido reduzida nesses modelos HFrEF e HFpEF, no modelo HFpEF para hipertensão, isso é compensado pelo aumento da fosforilação PLB (Fosfolamban é uma proteína capaz de modular a SERCA2A), mas não em HFrEF e HFpEF diabéticos, resultando em retardo da recaptação de Ca^2^ intracelular por SERCA.^8^

Um dos modelos animais mais usados para estudar a IC consiste na colocação de uma faixa constritiva de diâmetros variáveis ao redor da aorta ascendente.^9^ É importante ressaltar que a idade do animal, o diâmetro da banda e o posicionamento da constrição impactam no desfecho da doença. Isso provavelmente explica a variedade de fenótipos encontrados em estudos básicos disponíveis na literatura. Um dos primeiros estudos usando este modelo descreveu alterações morfológicas precoces no coração, o qual foi notável 2 meses após a bandagem aórtica com aumento de 80% do peso da massa do ventrículo esquerdo. A seguir, a literatura explorou vários aspectos da síndrome de IC usando esse modelo animal. Um dos modelos mais estudados neste cenário é o modelo do rato usando uma faixa de prata (0,6–0,7 mm de diâmetro interno) colocada 3 mm distante da raiz da aorta ascendente. Artigos anteriores encontraram mudanças estruturais no coração logo 3 semanas após o procedimento cirúrgico,^10^ e a gravidade da remodelação cardíaca aumentou ao longo do tempo. No entanto, os autores não exploraram as alterações estruturais do coração mais tardiamente no modelo experimental em seu estudo seminal. Como o estágio final da síndrome de IC pode variar desde os estágios iniciais, é importante compreender os mecanismos moleculares subjacentes envolvidos na fase crônica da IC induzida pela estenose aórtica.

A este respeito, o estudo recente de da Silva et al.,^11^ avançou nesse campo. Usando o mesmo modelo de rato descrito anteriormente, eles relataram que 28 semanas após a estenose aórtica em ratos foi encontrada na avaliação ecocardiográfica, atenuação significativa da fração de encurtamento e da fração de ejeção do ventrículo esquerdo, juntamente com o aumento do diâmetro diastólico do ventrículo esquerdo, um achado comum em vários modelos animais de IC.^12^ De fato, usando ensaio isolado do músculo papilar do ventrículo esquerdo, eles descobriram no ensaio de contração pós-repouso clássico que o músculo papilar tinha capacidade mecânica reduzida em comparação com o grupo de controle usando baixa concentração de cálcio extracelular. Uma vez que os níveis de cálcio foram aumentados, os valores foram semelhantes aos do grupo controle, sugerindo prejuízo na dinâmica do Ca ^2^ no grupo experimental, como é esperado durante a IC. Foi curioso que o nível de expressão do canal de cálcio do tipo L foi avaliado, ele foi encontrado aumentado no grupo experimental, o que não está de acordo com o ensaio do músculo papilar. No entanto, é importante destacar que proteínas auxiliares adicionais dão origem à função do canal de cálcio tipo L in vivo, e essas proteínas podem modular a função do canal.^13^ Assim, estudos funcionais usando a técnica de patch-clamp podem revelar esse resultado aparentemente contraditório. Além disso, os autores descobriram que o nível de expressão da principal bomba de extrusão citoplasmática de Ca^2^ encontrada nos cardiomiócitos, a SERCA2A, estava aumentada. No entanto, a proporção fosforilada de fosfolambam (PLB) na treonina-17 sobre o nível de PLB total foi atenuada. A PLB fosforilada em treonina-17 pode aumentar a atividade de SERCA2A. Assim, o resultado indica extrusão prejudicada do cálcio citoplasmático nos cardiomiócitos, como encontrado em outros modelos de IC.^12 , 14 , 15^ Na verdade, o resultado está bem alinhado com o aumento do tempo de decaimento do Ca^2^ avaliado em cardiomiócitos isolados carregados com experimentos fura-2AM. Portanto, independentemente da etiologia da insuficiência cardíaca, arritmias ventriculares são frequentemente observadas e refletidas em um distúrbio uniforme da homeostase do Ca^2^. O efeito bifásico observado da atividade SERCA sobre a propensão de ondas arritmogênicas de Ca^2^ adiciona detalhes importantes para um melhor entendimento da homeostase do Ca^2^ em diferentes modelos cardíacos.^8^

Assim, futuras investigações estudando o modelo animal na evolução temporal da doença podem contribuir significativamente para a compreensão dos mecanismos moleculares envolvidos no desenvolvimento da insuficiência cardíaca.
