# Supplementary material for: A Importância dos Estudos de Evolução Temporal Usando Modelos Experimentais de Doenças Cardíacas
Source: Arq Bras Cardiol. 2022 Feb 14;118(2):476–7. [Article in Portuguese] doi: 10.36660/abc.20210997 (PMC8856695; doi:10.36660/abc.20210997)
Supplement: Translation

# Translation: The Importance of Time-Course Studies Using Experimental Models of Cardiac Diseases

**Authors:** Diego Santos Souza, Danilo Roman-Campos

**Affiliations:** 1 Brazil Laboratório de CardioBiologia, Departamento de Biofísica, Escola Paulista de Medicina, Universidade Federal de São Paulo, São Paulo, SP – Brazil

**Keywords:** Cardiovascular Diseases, Heart Failure, Mortality, Health Care Costs, Ventricular Remodeling, Myoctes Cardiac, Rats

According to the American College of Cardiology, Heart Failure (HF) Syndrome is defined as a complex clinical syndrome that results from any structural or functional impairment of ventricular filling or ejection of blood.^1^ The etiological agent of the clinical syndrome of HF may result from different causes, which impact the prognosis and impose the necessity for specific treatment strategies, underlining the relevance of specific data elements for emphasizing these differences in HF, including the time course evolution of HF.^2^ This view is similar to the current Brazilian guideline for HF.^3^ Despite improvement in HF treatment and management, with a reduction in overall mortality rate over time,^4^ the death number and economic costs are still high. In 2014 a deep revision including 197 countries showed that HF’s overall global economic cost in 2012 was estimated at $108 billion per annum.^5^ Importantly, HF spending was largely different in high-income, middle, and low-income countries.^5^ For example, between 2014-2020, the annual median total medical costs for heart failure care were estimated at $24,383 per patient in the United States of America,^6^ meanwhile in Brazil in 2015, the average cost per patient with HF was 1,569 US$, taking into account the exchange rate in 2015.^7^ The high cost per patient is probably related to the lack of effective therapies to improve human health during HF. Then unveiling the molecular basis of cardiac remodeling found in the disease is one of the main challenges in cardiovascular medicine.

In the last years, significant advances in understanding molecular mechanisms of adaptive and maladaptive hypertrophy and heart failure in response to stress signals have shown that many extracellular factors and signaling pathways are involved in the remodeling of cardiomyocytes, the working cells found in the heart tissue. Since the time course of HF is etiological-dependent, it is fundamental to understand how the HF syndrome develops. For example, the intracellular dynamics of Ca^2^are modified in the different etiologies of HF with reduced (HFrEF) or preserved (HFpEF) ejection fraction. In animal models of diabetic or hypertensive HFpEF and HFrEF, studies have shown that magnitude and kinetics of Ca^2^release remain unchanged in both HFpEF models but impaired in HFrEF. Although Sarco/endoplasmic reticulum Ca^2^adenosine triphosphatase-2a (SERCA) protein density was reduced in these HFrEF and HFpEF models, in the HFpEF model for hypertension, this is offset by increased PLB phosphorylation (Phospholamban is a protein able to modulate SERCA2A), but not in diabetic HFrEF and HFpEF resulting in delay intracellular Ca^2^reuptake by SERCA.^8^

One of the most used animal models to study HF relies on placing a constricting band of variable diameters around the ascending aorta.^9^ It is important to highlight that animal age, the diameter of the band, and positioning of the constriction impact the outcome of the disease. It probably explains the variety of phenotypes found in basic studies available in the literature. One of the first studies using this model described early morphological changes in the heart, which was remarkable 2 months after aortic banding with 80% of left ventricular mass weight increment. Following, the literature explored several aspects of HF syndrome using this animal model. One of the most studied models in this setting is the rat model using a silver band (0.6–0.7 mm internal diameter) placed 3 mm around the root of ascending aorta. Previous reports had found structural changes in the heart as early as 3 weeks after the surgery procedure,^10^ and the severity of heart remodeling increased over time. However, the authors did not explore the structural heart changes at a later time in their seminal study. Since the end stage of HF syndrome may vary from the initial stages, it is important to understand the underlying molecular mechanisms involved in the chronic phase of HF induced by aortic stenosis.

In this regard, the recent study by da Silva et al.^11^ advanced in that field. Using the same rat model previously described, they reported that 28 weeks after aortic stenosis in rats was found in the echocardiographic evaluation, significant attenuation of fractional shortening and ejection fraction of the left ventricle, along with the enhanced left ventricular diastolic diameter, a common finding in several animal models of HF.^12^ Indeed, using isolated left ventricular papillary muscle assay, they found in the classical post rest contraction assay that papillary muscle had reduced mechanical capacity compared to the control group using low extracellular calcium concentration. Once the calcium levels were enhanced, the values were like the control group, suggesting impairment in Ca^2^dynamics in the experimental group, as expected during HF. Intriguing, once the expression level of the L-type calcium channel was evaluated, it was found enhanced in the experimental group, which is not in accordance with papillary muscle assay. However, it is important to highlight that additional auxiliary proteins give rise to the function L-type calcium channel in vivo, and these proteins can modulate the channel function.^13^ Thus, functional studies using the patch-clamp technique could uncover this apparently contradictory result. Also, the authors found that the expression level of the main cytoplasmic Ca^2^extrusion pump found in cardiomyocytes, SERCA2A, was increased. However, the phospholamban (PLB) phosphorylated ratio at threonine-17 over the total PLB level was attenuated. PLB phosphorylated at threonine-17 can enhance SERCA2A activity. Thus, the result indicates impaired extrusion of cytoplasmic calcium in cardiomyocytes, as found in other HF models.^12 , 14 , 15^ Indeed, the result is well-aligned with increased time to Ca^2^decay evaluated in isolated cardiomyocytes loaded with Fura-2AM experiments. Therefore, regardless of the etiology of heart failure, ventricular arrhythmias are frequently observed and reflected in a uniform disturbance of Ca^2^homeostasis. The observed biphasic effect of SERCA activity on the propensity of arrhythmogenic Ca^2^waves add important details for a better understanding of Ca^2^homeostasis in different heart models.^8^

Thus, future investigation studying the animal model later in the time course of the disease could significantly contribute to the understanding of the molecular mechanisms involved in the development of heart failure.
